# Molecular Characterization and Genetic Significance of a Rare Type-I Triallelic Pattern Involving a 15.3 Microvariant at the D19S433 Locus

**DOI:** 10.3390/ijms27156621

**Published:** 2026-07-24

**Authors:** Vera Djeliova, Stanislava Dimitrova-Nikolova, Tzanko Markov, Yanko Kolev, Atanas Hristov, Milka Mileva, Aleksandar Apostolov

**Affiliations:** 1 Department of the Molecular Biology of the Cell Cycle, Institute of Molecular Biology Acad. Rumen Tsanev, Bulgarian Academy of Sciences, “Acad. G. Bonchev” Street, Bl. 21, 1113 Sofia, Bulgaria; vera@bio21.bas.bg; 2 DNA Laboratory, National Institute of Forensics, Bul. Alexander Malinov 1, 1756 Sofia, Bulgaria; stasidbg@gmail.com (S.D.-N.); tzanko_markov@abv.bg (T.M.); 3 Department of Forensic Medicine, Multidisciplinary Hospital for Active Treatment “Gabrovo” District Hospital, 5300 Gabrovo, Bulgaria; drforensic@gmail.com; 4 Clinic of Forensic Medicine and Deontology, University Hospital Alexandrovska, 1 St. Georg Sofiiski Blvd., 1431 Sofia, Bulgaria; ahristov@medfac.mu-sofia.bg; 5 Department of Forensic Medicine and Deontology, Faculty of Medicine, Medical University, 2 Zdrave St., 1431 Sofia, Bulgaria; 6 Department of Virology, Institute of Microbiology, Bulgarian Academy of Sciences, 26 Acad. G. Bonchev Str, 1113 Sofia, Bulgaria

**Keywords:** D19S433, triallelic pattern, STR profiling, microvariant 15.3, Bulgarian population

## Abstract

Short tandem repeat (STR) analysis is the main reference standard in forensic genetics. Despite its reliability, rare genetic anomalies like triallelic patterns and microvariants challenge profile interpretation. This study characterizes an exceptional genetic finding identified during routine casework at the Research Institute of Forensic Science in Sofia, Bulgaria. DNA was isolated from dental remains of an unidentified male corpse and amplified using the Investigator^®^ ESSplex SE Plus kit targeting 15 autosomal STR loci. A well-balanced male genotype was obtained across 14 loci; however, a rare triallelic anomaly emerged at D19S433 (19q12). Three distinct peaks were identified: standard alleles 13 and 14, and an off-ladder allele at 261.09 bp, designated as microvariant 15.3. Quantitative peak height analysis showed that the combined intensity of alleles 13 and 14 (~1316 RFU) was approximately equal to that of microvariant 15.3 (1054 RFU). This balance confirms a Type I triallelic configuration, indicative of a localized somatic mutation or segmental duplication rather than trisomy or DNA mixture. Global databases (NIST STRBase and STRidER) and regional Bulgarian data confirmed that this specific 13/14/15.3 combination has not been previously documented. Documenting rare STR variations is critical to enriching genomic databases and ensuring maximum accuracy in forensic interpretations.

## 1. Introduction

Short tandem repeat (STR) analysis of highly polymorphic loci in the non-coding regions of the human genome is the standard method for individual identification and kinship testing in forensic genetics. STR profiling typically detects one or two alleles at each locus, corresponding to homozygous or heterozygous genotypes. Rarely, genetic anomalies such as null alleles, off-ladder (OL) alleles, microvariants, triallelic patterns, and mutations may occur [1]. Because these variants are uncommon, their documentation is important for accurate DNA profile interpretation. The National Institute of Standards and Technology (NIST) maintains the STRBase database [2], established in 1997 to catalog newly identified STR variants and anomalies [3]. Commercial STR kits include allelic ladders containing the most common alleles [4], but newly discovered variants require regular updates. Therefore, forensic geneticists should be aware of rare allelic variants, triallelic patterns, and mutations during STR interpretation [5]. Triallelic patterns are a locus-specific form of copy number variation (CNV) and are classified as Type I or Type II [6]. Type I, the more common form, is characterized by two alleles whose combined peak heights equal that of the third allele and is thought to result from a somatic mutation at a heterozygous locus [7]. It has been reported at frequencies of 0.04–0.05% in population studies [7,8,9], whereas triallelic STR profiles occur in approximately 1 in 1000 samples [1]. To date, about 401 autosomal triallelic STR patterns have been reported in STRBase [2]. However, data from the Balkan region, including Bulgaria, remain limited.

The main objective of this study is to present and analyze an extremely rare case of a Type I triallelic pattern at the autosomal STR locus D19S433, identified during routine expert work. The study focuses on the genetic characterization of the newly discovered allelic microvariant 15.3 and on documenting this unique triallelic combination, which has not yet been entered into international databases [2], thereby contributing to greater accuracy in the interpretation of complex DNA profiles in forensic practice.

## 2. Results

### 2.1. Case Context, Quality Control, and Initial STR Profiling

**Figure 1:**
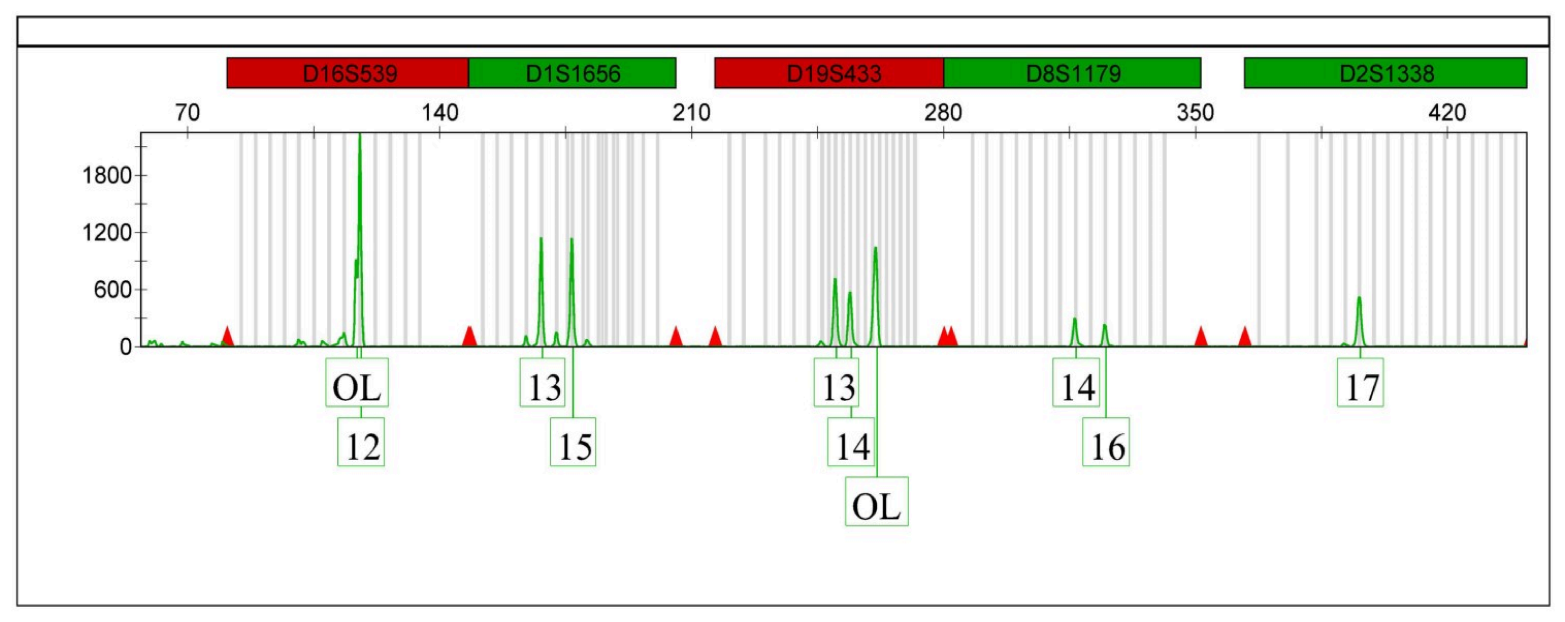
Electrophoregrams of part of the loci from the D19S433 locus and four other loci near it are presented. Amplification was performed using the Investigator^®^ ESSplex PCR Kit (QIAGEN, Hilden, Germany). The separation and size determination of the resulting PCR products were performed on an automatic sequencer, the 3130xl Genetic Analyzer. The results were analyzed using Gene Mapper™ v1.2 Full Software for human identification (HID) analysis.

**Figure 2:**
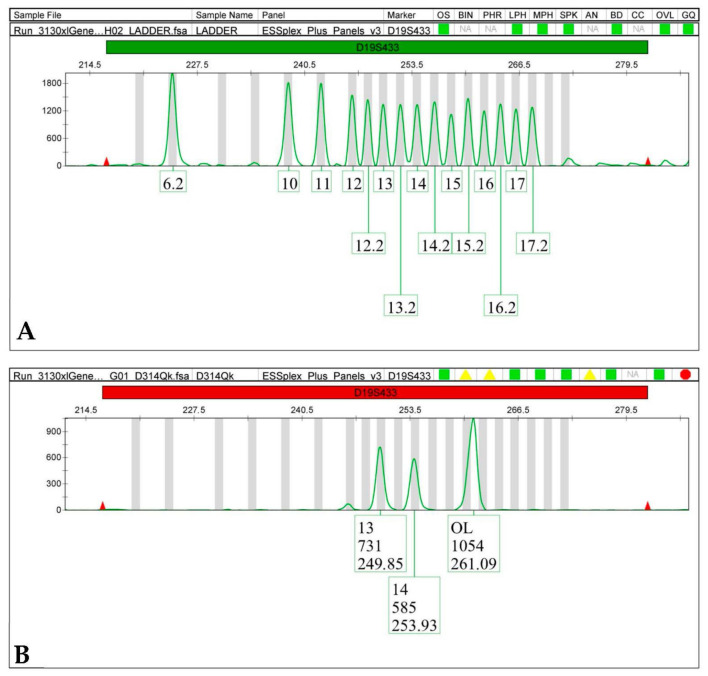
(**A**) Allelic Ladder from the Investigator^®^ ESS plex PCR Amplification Kit (QIAGEN) in D19S433 locus. The locus contains 20 established bins (gray bars) and 15 alleles, present in the allelic ladder: 6.2, 10, 11, 12, 12.2, 13, 13.2, 14, 14.2, 15, 15.2, 16, 16.2, 17, 17.2 (**B**) Three alleles in D19S433 locus from the DNA profile from the teeth from an unidentified male corpse are presented. Gray bars indicate established physical bins (alleles physically present in the ladder). Peaks falling within gray are automatically designated by the analysis software: 13, 14. The white zones indicate regions outside of established marker ranges; peaks falling within these areas are classified as Off-Ladder (OL) alleles. The following are indicated: Allele (Repeat #), RFU and PCR product sizes [bp] of observed alleles. Data validation was performed according to the REFERENCE MANUAL Gene Mapper™ v1.2 Full Software.

This study details a rare genetic anomaly identified during routine forensic casework at the DNA Laboratory of the National Institute of Forensic Science, which has held BDS EN ISO/IEC 17025 accreditation since 2014. The abnormality was discovered at the D19S433 locus in a DNA profile derived from the teeth of an unidentified male corpse. Following DNA extraction from two teeth and subsequent concentration of the DNA extract, two independent amplifications were performed using the Investigator^®^ ESSplex PCR Kit (QIAGEN, Hilden, Germany), yielding identical results. One representative STR profile from these amplifications is presented in this study (Figure 1 and Figure 2). According to the manufacturer’s data, the probability of identity (PI) for this specific multi-locus combination within the European population is approximately PI = 1.6 × 10^−19^. The integrity of the results was ensured by a confirmed control DNA genotype and the absence of amplification products in the negative control (reagent blank). The analysis yielded a well-balanced male DNA profile characterized by a triallelic abnormality at the D19S433 locus (Figure 1). Three distinct alleles were detected: 13, 14, and a third Off-Ladder (OL) allele with a length of 261.09 bp. While alleles 13 and 14 aligned with the standard bins of the allelic ladder included in the Investigator^®^ ESS plex PCR kit, the OL allele fell within a ‘white bar’ region, remaining unrecognized by the analysis software (Figure 2, Table 1). The biological sample provided (2 teeth) was completely consumed during the analysis. In addition, the teeth originate from the skeletal remains of an unidentified person who was buried. Therefore, additional experiments such as amplification with a different STR analysis kit and whole-genome sequencing of the sample were not performed.

### 2.2. Characterization, Sizing, and Nomenclature of the 15.3 Microvariant

The D19S433 locus is situated on the long arm of chromosome 19 (19q12) at the physical position of 35.109 Mb. It is characterized as a compound tetranucleotide repeat with the motif [CCTT]_n_ ccta [CCTT]_n_ cttt [CCTT]_n_ [2,3,10]. Allelic variations at this locus typically range from 5.2 to 20 repeats [1]. The observed alleles in this case follow this documented motif, where alleles 13 and 14 correspond to the standard ladder, while the 15.3 microvariant represents a rare insertion/deletion event. To ensure accuracy, the result was confirmed through repeat amplification. The detected OL fragment (261.09 bp) is positioned between allele 15.2 (260.30 bp) and allele 16 (262.25 bp). Given that the length of this fragment is exactly one base pair longer than the preceding allele and one base pair shorter than the following full repeat, the microvariant was designated as 15.3. Based on these fragment size data, we concluded that the anomaly represents a rare triallelic pattern involving alleles 13, 14, and 15.3. The fragment sizes and repeat numbers were verified according to the GenBank Accession GRCh38 reference genome and the STR nomenclature guidelines proposed by Phillips et al. [10], using sequence information from NIST STRBase, Accession PRJNA380574 [2,3]. The D22S1045 locus consists of a tetranucleotide repeat structure: [CCTT]_n_ ccta [CCTT]_n_ cttt [CCTT]_n_ [2,3,10].

**Table 1:** PCR product sizes and Relative Fluorescence Units (RFU) of observed alleles at the D19S433 locus. The locus is situated at chromosome 19q12 and consists of a tetranucleotide tandem repeat motif: [CCTT]_n_ ccta [CCTT]_n_ cttt [CCTT]_n_ (GenBank Accession: GRCh38; Phillips C. et al. [10]).

Allele (Repeat #)	RFU	PCR Product Sizes of Observed Alleles [bp]
13	731	249.85
14	585	253.93
OL (15.3)	1054	261.09

### 2.3. Evaluation of Global and Regional Population Databases

A search of the NIST STRBase [2,3] revealed that while 52 allelic variants have been documented for the D19S433 locus, microvariant 15.3 has been reported only once previously—by Tara Roy of the Washington State Police Crime Lab. Although that study recorded a fragment size of 127.09 bp using an ABI 3130xl sequencer (Applied Biosystems, Japan, SN 18234-023) and the AmpFLSTR^®^ Identifiler™ kit (Thermo Fisher Scientific, Foster City, CA, USA) the allelic designation remains consistent with our findings. The discrepancy in base pair (bp) size is expected, as absolute fragment lengths vary between different PCR amplification kits due to the use of distinct primer sets. Furthermore, an investigation of the STRidER database [11] showed that variant 15.3 was not detected in an allele frequency analysis encompassing 10,073 individuals from 20 population groups (7073 from Europe and 3000 from Asia). Similarly, this microvariant was absent in a comprehensive meta-analysis of 63 population groups conducted by Elzalabany et al. [12]. Locally, a recent population study of 500 Bulgarian individuals using the NGM Detect™ kit also failed to detect the 15.3 microvariant at the D19S433 locus [13], further highlighting the extreme rarity of this allele within both regional and global contexts.

### 2.4. Cross-Reference Analysis with Commercial STR Multiplex Systems

To further characterize the 15.3 microvariant, which resides outside the standard bins of the Investigator^®^ ESS plex kit, several contemporary commercial STR multiplex systems were cross-referenced. According to the NGM Detect™ PCR Amplification Kit manual (2017), a similar 15.3 allele at the D19S433 locus was neither detected in the reference populations nor included in the kit’s allelic ladder. Similarly, the 15.3 variant was absent from the allelic ladder of the PowerPlex^®^ Fusion 6C System (Promega). This is notable as the PowerPlex^®^ Fusion 6C is a high-resolution system with one of the most significant discrimination powers currently available, featuring a probability of identity (PI) of 9.09\times 10^−31^ [14]. The absence of this microvariant in such widely used, high-density kits further underscore its extreme rarity.

### 2.5. Quantitative Peak Intensity Analysis and Pattern Classification

The three identified alleles—13, 14, and 15.3 (OL)—exhibited distinct peak intensity characteristics. The 15.3 microvariant showed the highest intensity at 1054 RFU, while alleles 13 and 14 recorded intensities of 731 RFU and 585 RFU, respectively. Notably, the combined intensity of alleles 13 and 14 (~1316 RFU) is approximately equivalent to the peak height of the detected microvariant (Table 1). The peak height data confirm a Type I triallelic pattern. As shown in Table 1, the sum of the intensities of alleles 13 (731 RFU) and 14 (585 RFU) is 1316 RFU, which is approximately equivalent to the intensity of the 15.3 microvariant (1054 RFU). The slight deviation in peak height ratios is within the expected quantitative variation in capillary electrophoresis and is consistent with a Type I triallelic pattern. Based on these Relative Fluorescence Unit (RFU) ratios, the observed triallelic state is classified as a Type I pattern, which is the more prevalent form of this anomaly. This balance is characteristic of a somatic mutation or localized duplication rather than a trisomy or DNA mixture. A review of the NIST STRBase indicates that while 19 triallelic patterns have been documented for the D19S433 locus to date [2,3], the specific combination of alleles 13, 14, and 15.3 has not been previously reported.

## 3. Discussion

In this study, we present a rare genetic finding identified during routine forensic casework: a triallelic abnormality at the D19S433 locus, where one of the three detected alleles represents a rare microvariant (15.3).

Short Tandem Repeat analysis is currently the primary reference standard for human DNA identification. While the method is highly standardized and robust, the occurrence of genetic anomalies can occasionally complicate the interpretation of DNA profiles. Such complications in STR genotyping typically arise from allelic microvariants, so-called off-ladder (OL) alleles, null alleles, triallelic states, or de novo mutations [1,3,15].

Novel allelic variants that are not represented in the standard allelic ladder may result from the insertion or deletion of full repeat units, single-base indels, or partial repeats, leading to the formation of microvariants [5,16,17]. Continuously updating and enriching commercial PCR amplification kits with these rare variant alleles is essential for ensuring the accuracy and reliability of forensic DNA profiling.

The detection of triallelic patterns at STR loci is a rare yet critical occurrence in forensic genetics. In the present study, analysis of a sample derived from an unidentified cadaver revealed a triallelic profile at the D19S433 locus, consisting of alleles 13, 14, and the 15.3 microvariant. Notably, this anomaly was isolated to this specific locus, with all other 14 examined autosomal STR loci displaying standard biallelic or homozygous genotypes. This locus-specific observation effectively rules out the hypotheses of a DNA mixture or sample contamination, confirming the result as a true genetic triallelic variant.

To elucidate the mechanisms underlying triallelic conditions, several studies involving inheritance tracing and sequencing have been conducted [18,19,20,21,22,23,24]. These genetic anomalies typically arise from allelic duplication within the same or a different chromosome [18,21,24], chromosomal rearrangements [19], or, more rarely, through mosaicism, chimerism, or trisomy [25,26,27]. Recent genomic sequencing has further revealed that copy number variants (CNVs) are significantly more prevalent than previously estimated [22,23]. In tetranucleotide STR loci, the vast majority of mutational events involve the gain or loss of a single repeat unit [6,28]. This process is widely attributed to replication slippage (strand slippage), where DNA polymerase dissociates and misaligns on the lagging strand during replication [28]. While unique genomic sequences exhibit a low mutation rate (approx. 10^−9^ nucleotides per generation), the repetitive nature of STRs increases this probability by several orders of magnitude, reaching rates between 10^−6^ and 10^−2^ [29]. For a triallelic state to manifest constitutively across an individual’s tissues, such a mutational event must occur during mitosis in the early stages of embryonic development, specifically between the blastocyst and germ layer phases [6,30,31].

In the present investigation, the triallelic pattern was identified during the routine DNA profiling of unidentified human remains. A significant limitation encountered was the complete consumption of the available biological material during the initial analytical procedures, which precluded further confirmatory testing, such as Next-Generation Sequencing (NGS) or additional specialized PCR amplifications [31,32]. Furthermore, as no biological relatives have been identified to date, it was not possible to conduct a pedigree analysis to verify the heritability of this specific triallelic variant.

Based on data from research by leading teams worldwide [6,33], our results suggest a plausible hypothesis for an integrated model for the emergence of the phenotype observed in the present study. Rather than considering events in isolation, we suggest that the 13/14/15.3 triallelic pattern results from a cascade of genetic events driven by the structural specificity of the locus [1,34] and its chromosomal environment [6,35].

The primary step in the etiology of the observed phenotype is likely to involve structural rearrangements at the chromosomal level. Chromosome 19 is distinguished by a unique architecture, with the highest gene density in the human genome and an extremely high density of repeated elements, particularly from the Alu-repeat family [33,35]. These short interspersed nuclear elements (SINEs) serve as a critical substrate for non-allelic homologous recombination (NAHR), a process mediated by highly homologous sequences that predispose to misalignment during meiosis or early mitosis.

It is highly likely that a localized segmental duplication occurs, encompassing the D19S433 locus (located in the 19q12 region). This type of copy number variation (CNV) leads to the formation of paralogous copies of the locus within the same chromosomal region [7,8]. The presence of these low-copy repeats provides a biological explanation for the transition from the standard biallelic state to the overt triallelic configuration [3,36].

The next step in our hypothesis involves the differentiation of the duplicated copies by micromutations. The high mutation rate at the D19S433 locus makes it highly susceptible to errors during DNA replication. We hypothesize that, after the duplication, an insertion or deletion occurred at the nucleotide level via slipped-strand mispairing [34,36]. This specific event generated the rare microvariant 15.3 (261.09 bp), which deviates from the standard tetranucleotide repeats for this locus. The presence of such off-ladder alleles is often indicative of dynamic mutational processes occurring in complex repetitive sequences [7,37].

The final result of these processes manifests as a Type I trialselelic pattern. The quantitative distribution of the fluorescent signal (where the sum of the intensity of alleles 13 and 14 corresponds to the intensity of allele 15.3) is strong evidence for a somatic mutational origin [6,38]. This quantitative balance suggests that the mutation cascade occurred at an early stage of embryogenesis, leading to a stable cell lineage that carries the three different alleles and is fully represented in the hard tissues (teeth) of the individual [14,38].

Despite these constraints, the high quality of the obtained electropherograms, the balanced peak height ratios characteristic of a Type I pattern, and the consistency across all other autosomal loci provide strong evidence for the authenticity of the 13/14/15.3 triallelic genotype at the D19S433 locus. Such rare triallelic patterns and microvariants—including the fractional 15.3 allele—are critical for expanding population databases, but their interpretation requires meticulous validation to rule out technical artifacts or primer-binding-site mutations [39]. Indeed, recent forensic literature highlights that genotyping discrepancies at the D19S433 locus, particularly between capillary electrophoresis (CE) and massively parallel sequencing (MPS/NGS), are often driven by flanking region deletions or point mutations that can lead to primer binding failures and the appearance of “silent” or null alleles [40,41]. While MPS technologies offer enhanced discrimination power and sequence-based resolution to deconstruct these complex genomic architectures [42], this case underscores the challenges inherent in forensic casework involving degraded or limited samples, where rare genetic anomalies must be interpreted with caution yet documented to aid future identification efforts [39,40,41,42].

## 4. Materials and Methods

### 4.1. Sample Extraction and DNA

As a sample for DNA profiling at the DNA laboratory of the National Institute of Forensic Science, the investigating authorities provided 2 teeth taken from an unidentified corpse of a man. The teeth were pulverized in a cryogenic mill. DNA extraction was performed with the QIAamp® DNA Micro Kit (Qiagen, Hilden, Germany) according to the manufacturer’s protocol.

### 4.2. DNA Amplification

DNA amplification was performed with 1 ng of DNA from the DNA isolate. The PCR reaction was performed with the Investigator^®^ ESS plex PCR Amplification Kit (QIAGEN, Hilden, Germany) containing 15 STR loci: TH01, D3S1358, vWA, D21S11, D16S539, D1S1656, D19S433, D8S1179, D2S1338, D10S1248, D22S1045, D12S391, FGA, D2S441, D18S51 and the sex-determining system—amelogenin on a PCR Gene Amp PCR System 9700 (Applied Biosystems, Foster City, CA, USA) according to a preset program of 30 cycles, according to the manufacturer’s protocol.

### 4.3. Electrophoresis and Analysis

The separation and size determination of the resulting PCR products were performed on an automatic sequencer, the 3130xl Genetic Analyzer for Human Identification (Applied Biosystems, Tokyo, Japan, SN 18234-023). The analysis used capillary electrophoresis (POP-4™ Polymer, (Shizuoka, Japan) with laser detection of DNA fragments in the presence of an internal standard, SST-BTO550.

### 4.4. Data Analysis and Validation

The results were analyzed using Gene Mapper™ v1.2 Full Software (Applied Biosystems) for human identification (HID) analysis. Control and standardization of the obtained results were ensured through: (i) Positive control (DNA control 007) and negative control (NC); (ii) Internal standard (SST-BTO 550) and an allelic control for the respective STR markers contained in the Investigator^®^ ESS plex PCR Amplification Kit. Data validation was performed according to the REFERENCE MANUAL Gene Mapper™ v1.2 Full Software.

## 5. Conclusions

In this study, we present a case of a triallelic state at the D19S433 locus, belonging to the more common type I triallelic pattern. One of the three detected alleles is located outside the allelic groups of witnesses and can be represented as a confirmed new allelic microvariant (15.3) for the D19S433 locus. To our knowledge, it has not yet been mapped in the latest STR DNA analysis kits. Although the frequency of such anomalies is extremely low, the possibility of their detection in standard DNA analysis cannot be ruled out. The detection of triallelic states or rare allelic microvariants increases the discriminatory power in identifying a given individual, for example, in some specific criminal investigations, in proving the origin of a biological trace to a specific person, or in determining a family relationship. The results obtained by our team could serve as guidance for DNA experts on the likelihood of similar findings in STR DNA profiling and suggest an approach for interpreting such data in preparing an expert opinion.

## Data Availability

The data used or analyzed in this study are available from the first author Vera Lyubchova Djeliova.
